# Phytase expressed from *Saccharomyces pombe* ameliorates footpad lesions in cage‐reared broiler chicks

**DOI:** 10.1002/vms3.745

**Published:** 2022-02-26

**Authors:** De Xin Dang, Seong Guk Chun, In Ho Kim

**Affiliations:** ^1^ Department of Animal Resource & Science Dankook University Cheonan South Korea

**Keywords:** broiler chick, footpad lesion, nutrient digestibility, phytase, toe ash

## Abstract

**Background/aim:**

The condition of footpad is an important aspect of poultry welfare. This is a problem that plagues the poultry industry because it occurs whether birds are reared in the cage or on the floor. It is reported that feeding phytase to floor‐reared broiler chicks could ameliorate footpad lesions, which is related to the reduction of litter moisture. However, some studies reported that phytase supplementation could ameliorate footpad lesions, but did not affect litter quality. Therefore, phytase supplementation may have other potential mechanisms to improve the footpad lesions. Cage‐reared broiler chicks were used in this study because they had no access to litter.

**Material and methods:**

A total of 234 1‐day‐old broiler chicks were randomly assigned to three groups based on the initial body weight (42.22 ± 0.18 g) with six replicate cages and 13 birds (mixed sex) per cage. The experimental period was 45 days. Dietary treatments were based on a corn–soybean meal‐basal diet and supplemented with 500 and 750 FTU/kg *Saccharomyces pombe* expressed phytase. The unit of phytase (FTU) was defined as the amount of enzyme that catalyzes the release of one micromole phosphate from phytate per minute at 37°C and pH 5.5.

**Result and conclusion:**

We found that dietary supplementation of *S. pombe* expressed phytase could improve calcium and phosphorus digestibility and subsequent improvement in toe ash, thus ameliorating footpad lesions in broiler chicks with no access to litter.

## Abstract 2

The condition of footpad is an important aspect of poultry welfare. We tested the efficacy of phytase by investigating the apparent ileal digestibility, growth performance, and toe ash, so as to reveal the relationship between phytase supplementation and footpad lesions amelioration.

## 1 INTRODUCTION

Footpad quality is an important aspect of poultry welfare. When the lesion situation is serious, it will cause pain in birds, thus impairing the feed intake and growth performance. In addition, chicken paw prices have escalated due to an insatiable demand for high‐quality paws in export markets. The lesions are a concern to the poultry industry as a cause of product downgrade issues (Shepherd & Fairchild, 2010). The event of footpad lesions occurs in both cage‐reared or floor‐reared broiler chicks (Roenchen et al., 2007). It has been reported that dietary supplementation of phytase was a way to ameliorate the footpad dermatitis in floor‐reared broiler chicks, which was related to the prevention of wet litter (Shepherd & Fairchild, 2010). However, Delezie et al. (2015) noted that the mechanisms of footpad lesions amelioration by supplementing phytase may have other aspects besides improving litter quality. Since the cage‐reared broiler chicks had no access to litter, we want to understand better whether dietary supplementation of phytase improves footpad lesions when the factor of litter quality is eliminated.

In addition, the digestibility of phosphorus and calcium, growth performance, and bone ash are commonly used parameters to evaluate the efficacy of phytase (Dersjant‐Li et al., 2015). We tested the efficacy of phytase by investigating the apparent ileal digestibility, growth performance, and toe ash, so as to reveal the relationship between phytase supplementation and footpad lesions amelioration. We hypothesized that dietary supplementation of *Saccharomyces pombe* expressed phytase could improve the apparent ileal digestibility and subsequent improvement in toe ash and growth performance, thus improving footpad lesions in cage‐reared broiler chicks.

## 2 MATERIALS AND METHODS

### 2.1 Information of phytase

The *Escherichia coli* gene‐origin phytase expressed by *Schizosaccharomyces Pombe* (ATCC 5233) used in this study (Phyzyme XP; Danisco Animal Nutrition, Marlborough, Wiltshire, UK) was formed in a fine granular. According to the European Food Safety Authority (EFSA, 2019), the stability of this phytase is over 95% after storage in 20°C environment for 6 months. The optimal pH is 4.5.

One phytase unit (FTU) was defined as the amount of enzyme that catalyzes the release of one micromole phosphate from phytate per minute at 37°C and pH 5.5 (EFSA, 2019).

### 2.2 Animals and housing

A total of 234 1‐day‐old Ross 308 broiler chicks were randomly assigned to three groups based on the initial body weight (42.22 ± 0.18 g). There were six replicate cages per treatment and 13 birds (mixed sex) per cage. The size of cage was 1.55 × 0.75 × 0.55 m. All birds were housed in a three‐floor battery cages. The temperature of room was 32°C at start and reduced by 2°C per week up to 24°C. The relative humidity of room was 65%. During days 1–7, birds were provided light for 24 h. During days 8–45, birds were provided light for 16 h and dark for 8 h. The intensity of light was about 10 lux. Two feeders and two nipple drinkers were equipped in the cage to provide feed and water ad libitum to birds.

### 2.3 Treatments and diets

The experiment lasted for 45 days and was divided into four phases: starter, days 1–10; grower, days 11–24; finisher 1, days 25–38; and finisher 2, days 39–45. Dietary treatments were based on a corn–soybean meal basal diet (Table 1) and supplemented with 500 and 750 FTU/kg phytase. Phytase was mixed with 1 kg of feed by hand, and then premix was mixed with the remaining feed by using a blender to ensure homogeneity. Diets were formulated to meet the nutrient requirements recommended by the Aviagen (2014) nutrition guide for Ross 308 and provided in mash form.

**TABLE 1:** Composition and nutrient levels of the experimental basal diet (%, as‐fed basis)

	Feeding phases			
	Starter (days 0–10)	Grower (days 11–24)	Finisher 1 (days 24–38)	Finisher 2 (days 38–45)
Ingredients, %				
Corn	50.76	52.61	56.41	57.09
Soybean meal, 48%	34.53	27.54	23.62	22.69
Canola meal	5.00	5.00	5.00	5.00
Brown rice	5.00	10.00	10.00	10.00
Yellow grease	1.71	1.99	2.37	2.91
Tricalcium phosphate	0.82	0.67	0.38	0.22
Limestone	0.86	0.91	0.95	0.87
Vitamin and trace mineral premix^1^	0.35	0.35	0.35	0.35
DL‐Methionine, 99%	0.30	0.31	0.27	0.23
Salt	0.24	0.24	0.27	0.28
l‐Lysine HCl	0.21	0.17	0.17	0.16
l‐Threonine, 98.5%	0.09	0.08	0.08	0.07
Choline‐Cl, 60%	0.08	0.08	0.08	0.08
Sodium bicarbonate	0.05	0.05	0.05	0.05
Total	100.00	100.00	100.00	100.00
Calculated value, %				
Arginine	1.52	1.32	1.19	1.16
Lysine	1.38	1.16	1.06	1.03
Methionine	0.63	0.61	0.55	0.50
Methionine + Cysteine	1.00	0.94	0.87	0.81
Leucine	1.87	1.65	1.54	1.52
Isoleucine	0.99	0.85	0.77	0.75
Threonine	0.91	0.81	0.75	0.73
Valine	1.08	0.95	0.87	0.85
Available phosphorus	0.45	0.44	0.38	0.35
Analyzed composition, %				
Metabolizable energy, MJ/kg	12.60	12.92	13.20	13.39
Crude protein	23.13	20.24	18.65	18.22
Crude fat	4.61	4.98	5.45	6.00
Crude fibre	3.16	3.09	3.04	3.03
Dry matter	85.33	85.33	85.56	85.86
Crude ash	4.92	4.62	4.24	3.99
Calcium	0.90	0.85	0.75	0.66
Total phosphorus	0.81	0.81	0.74	0.71
Sodium	0.21	0.21	0.21	0.21
Phytate phosphorus	0.27	0.26	0.26	0.26
Digestible lysine	1.25	1.05	0.96	0.93
Digestible methionine	0.61	0.58	0.53	0.48
Digestible cysteine	0.31	0.28	0.27	0.26
Digestible sulphur amino acid	0.92	0.87	0.80	0.75
Digestible threonine	0.78	0.69	0.64	0.62
Digestible tryptophan	0.22	0.19	0.17	0.17
Digestible valine	0.95	0.81	0.74	0.72
Digestible leucine	1.71	1.48	1.38	1.36
Digestible isoleucine	0.88	0.74	0.67	0.65
Digestible arginine	1.39	1.17	1.06	1.03

^1^ Provided per kg of complete diet: 37.5 mg Zn (as ZnSO_4_); 37.5 mg Mn (as MnO_2_); 37.5 mg Fe (as FeSO_4_·7H_2_O); 3.75 mg Cu (as CuSO_4_·5H_2_O); 0.83 mg I (as KI); and 0.23 mg Se (as Na_2_SeO_3_·5H_2_O), 15,000 IU of vitamin A, 3750 IU of vitamin D_3_, 37.5 IU of vitamin E, 2.55 mg of vitamin K_3_, 3 mg of Thiamin, 7.5 mg of Riboflavin, 4.5 mg of vitamin B_6_, 24 μg of vitamin B_12_, 51 mg of Niacin, 1.5 mg of Folic acid, 0.2 mg of Biotin, and 13.5 mg of Ca‐Pantothenate.

### 2.4 Sample collection and measurements

#### 2.4.1 Feed composition analysis

After homogeneous mixing, about 250 g of feed samples from each treatment diet in each period were collected in triplicate. All feed samples were dried in a 70°C oven for 72 h. Then, feed samples were ground and sieved with a 1‐mm sieve. Feed composition analyses were conducted on these feed samples. According to the procedure established by the AOAC (2000), the dry matter (method 930.15), crude protein (nitrogen × 6.25; method 968.06), crude fat (method 954.02), crude ash (method 942.05), calcium (method 984.01), phosphorus (method 965.17), and crude fibre (method 991.43) levels in the diet were analyzed. Then, the representative feed samples in each group were hydrolyzed with 6 N HCl for 24 h at 110°C. An amino acid analyzer (2690 Alliance, Waters, Inc., Milford, MA, USA) was used for the determination of amino acid contents in the diet. Energy in feed was measured by a bomb calorimeter (Parr 6100; Parr Instrument Co., Moline, IL, USA). Phytate‐P in raw materials and diets was determined using the method described by Reichwald and Hatzack (2008). Absorbance was determined using a Media spectrophotometer (Marcel Lamidey S.A., Châtillon, France) at a 519 nm wavelength. Sodium was determined in accordance with AOAC (2005) using microwave plasma‐atomic emission spectrometry (4100 MP‐AES; Agilent Technologies, Santa Clara, USA).

#### 2.4.2 Growth performance

All birds were weighed on days 1, 11, 25, 39, and 45 to calculate body weight gain (BWG). Cage‐based feed intake (FI) was calculated daily. Feed conversion ratio (feed to gain ratio; FCR) was calculated based on the values of BWG and FI. Mortalities were collected and recorded daily.

#### 2.4.3 Apparent ileal digestibility

During days 38–45, 0.2% chromium oxide was supplemented to the diet in order to determine apparent ileal digestibility (AID) of crude protein, calcium, and phosphorus. At the end of the experiment, two birds were randomly selected from each cage and euthanized by cervical dislocation. A portion of the small intestine from Meckel's diverticulum proximal to the ileocecal junction was removed in order to collect ileal digesta samples for AID measurements. According to the procedure established by the AOAC (2000), the crude protein (nitrogen × 6.25; method 968.06), calcium (method 984.01), and phosphorus (method 965.17) composition in the digesta samples were analyzed. Chromium concentrations were determined by atomic absorption spectrophotometry (UV‐1201; Shimadzu, Kyoto, Japan). The apparent digestibility values for ileal nutrients were calculated as follows: CAID = 1 − [(ID × AF)/(IF × AD)], where CAID is the coefficient of apparent ileal nutrient digestibility; ID is the concentration of an indigestible marker in diet; AF is the nutrient concentration in ileal digesta; IF is the indigestible‐marker concentration in ileal digesta; and AD is the nutrient concentration in diet.

#### 2.4.4 Toe ash

The left and right middle toes were excised from the above euthanized birds and pooled separately to yield four samples of toes per replicate cage. These were averaged for the statistical analysis of the toe ash data. The composite samples were dried overnight at 100°C, extracted in ether for 6 h, and ashed in a muffle furnace for 18 h at 600°C.

#### 2.4.5 Footpad lesions score

Lesions score of footpad dermatitis was measured on day 44 for all birds. Footpad dermatitis was scored on a four‐point scale: score 0, no lesions on the footpads; score 1, small lesions of the footpad epithelium (< 1 cm); score 2, larger lesions (>1 cm); and score 3, dorsal swelling visible.

### 2.5 Statistical analysis

All data were statistically analyzed using the General Linear Model procedure (SAS Inst. Inc., Cary, NC, USA) in a completely randomized block design. The replicate cage was used as the experimental unit. Orthogonal contrasts were used to examine the linear and quadratic effects in response to increasing the dietary supplementation of phytase. Variability in the data was expressed as the standard error of means (SEM), and *p* < 0.05 was considered statistically significant.

## 3 RESULTS AND DISCUSSION

In the present study, dietary supplementation of *S. pombe* expressed phytase did not affect the growth performance of broiler chicks (Table 2). As reported by Manangi and Coon (2008), the development of growth performance was dose‐dependent with the increase of dietary phytase levels. They observed that feeding broiler chicks with 250, 500, 750, 1000, 1500, 2000, and 5000 FTU/kg *S. pombe* expressed phytase‐containing diet linearly increased the BWG and FI, whereas linearly decreased the FCR. Therefore, dietary supplementation of 250 and 500 FTU/kg *S. pombe* expressed phytase did not affect the growth performance of broiler chicks, which may be related to the lower levels of phytase used in this study.

**TABLE 2:** Effects of dietary supplementation of *S. pombe* expressed phytase on growth performance in cage‐reared broiler chicks

	*S. pombe* phytase, FTU/kg				*p*‐Value	
Items	0	500	750	SEM	Linear	Quadratic
IBW, g	42.21	42.22	42.22	0.178	0.996	0.988
BWG, g						
Days 1–10	250.72	253.35	255.59	3.452	0.339	0.964
Days 11–24	629.55	621.82	623.52	13.776	0.762	0.785
Days 25–38	1155.21	1161.22	1170.41	22.546	0.642	0.955
Days 39–45	621.09	626.10	635.30	8.528	0.262	0.845
Days 1–45	2656.57	2662.49	2684.81	22.201	0.386	0.768
FI, g						
Days 1–10	349.69	354.85	362.31	5.527	0.133	0.868
Days 11–24	1078.99	1098.10	1083.35	11.406	0.792	0.249
Days 25–38	2330.64	2358.68	2324.57	11.926	0.725	0.055
Days 39–45	1103.43	1124.69	1138.61	13.304	0.086	0.826
Days 1–45	4862.76	4936.32	4908.84	28.052	0.268	0.167
FCR						
Days 1–10	1.40	1.40	1.42	0.019	0.400	0.781
Days 11–24	1.72	1.77	1.74	0.047	0.707	0.454
Days 25–38	2.02	2.04	1.99	0.043	0.614	0.549
Days 39–45	1.78	1.80	1.79	0.014	0.477	0.536
Days 1–45	1.83	1.86	1.83	0.017	0.949	0.236
Mortality, %	0.08	0.12	0.12	0.029	0.279	0.525

Abbreviations: BWG, body weight gain; FCR, feed conversion ratio; FI, feed intake; IBW, initial body weight; SEM, standard error of the mean.

The mechanism of improving nutrient digestibility by phytase supplementation is generally accepted (Dang & Kim, 2021). Phytase could hydrolyze phytate and release phytate‐bound nutrient ingredients, thus improving the utilization of nutrients in animals feed ingredients (Delezie et al., 2015). In this study, broiler chicks fed the diet supplemented with *S. pombe* expressed phytase increased the AID of calcium (*p* = 0.036) and phosphorus (*p* = 0.045), but did not affect the crude protein (Table 3), which was affirmed by the study of Hajimohammadi et al. (2020). In brief, dietary supplementation of 250 and 500 FTU/kg *S. pombe* expressed phytase could degrade phytate, which was manifested in the increase of the AID of phosphorus and calcium.

**TABLE 3:** Effects of dietary supplementation of *S. pombe* expressed phytase on apparent ileal digestibility in cage‐reared broiler chicks

	*S. pombe* phytase, FTU/kg				*p*‐Value	
Items	0	500	750	SEM	Linear	Quadratic
Ca, %	56.11^b^	57.13^ab^	57.71^a^	1.332	0.036	0.896
P, %	46.55^b^	47.39^ab^	48.05^a^	1.944	0.045	0.968
CP, %	66.46	66.77	66.39	2.427	0.983	0.907

Abbreviations: Ca, calcium; CP, crude protein; P, phosphorus; SEM, standard error of the mean.

^a,b^ Different superscripts within a row indicate a significant difference (*p* < 0.05).

Toe ash is one of the sensitive parameters for the utilization of phosphorus and calcium (Garcia & Dale, 2006). In this study, dietary supplementation of *S. pombe* expressed phytase resulted in an increase of toe ash (*p* = 0.023; Table 4), which was affirmed by the study of Selle et al. (2009). Therefore, we considered that dietary supplementation of *S. pombe* expressed phytase could increase the utilization of calcium and phosphorus, which was manifested in the improvement of toe ash.

**TABLE 4:** Effects of dietary supplementation of *S. pombe* expressed phytase on toe ash and footpad lesions score in cage‐reared broiler chicks

	*S. pombe* phytase, FTU/kg				*p*‐Value	
Items	0	500	750	SEM	Linear	Quadratic
Footpad lesion score	2.25^b^	2.13^ab^	1.75^a^	0.707	0.044	0.943
Toe ash, %	11.35^b^	12.89^ab^	13.36^a^	0.571	0.023	0.454

Abbreviation: SEM, standard error of the mean.

^a,b^ Different superscripts within a row indicate a significant difference (*p* < 0.05).

Footpad quality is an important aspect of poultry welfare. It occurs in both floor‐reared or cage‐reared birds. It is reported that dietary supplementation of phytase could ameliorate the footpad lesions in floor‐reared broiler chicks, which was related to the prevention of wet litter (Shepherd & Fairchild, 2010). However, Delezie et al. (2015) reported that feeding floor‐reared broiler chicks with *Pichia pastoris* expressed phytase did not improve the litter quality, but ameliorate the footpad lesions. In this study, phytase supplementation also ameliorated the footpad lesions in cage‐reared broiler chicks (*p* = 0.044; Table 4) because cage‐reared broiler chicks had no access to litter. Therefore, we considered that the mechanism of improving footpad lesions by phytase supplementation has other aspects instead of the litter quality improvement. Mukovozov et al. (2021) noted that atopic dermatitis in humans was related to poor bone health. In addition, poor absorption of certain minerals could also cause dermatitis in domestic animals (Soetan et al., 2010). The footpad lesions ameliorated by phytase supplementation have been reported to correspond to the improvement of bone quality and phosphorus and calcium digestibility (Shepherd & Fairchild, 2010). Therefore, we considered that the footpad lesions improved by *S. pombe* expressed phytase supplementation were related to the improvement of phosphorus and calcium digestibility and toe ash.

Therefore, *S. pombe* expressed phytase supplementation has great significance for ameliorating footpad lesions in cage‐reared broiler chicks, which was related to the increase of phosphorus and calcium utilization, manifested in the increase of phosphorus and calcium digestibility and subsequent improvement in toe ash.

## CONFLICT OF INTEREST

The authors declare no conflict of interest.

## ETHICS STATEMENT

This experimental protocol was approved by the Animal Care and Use Committee of Dankook University (DK‐1‐1706).

## AUTHOR CONTRIBUTIONS

*Writing—original draft, investigation, writing—review & editing*: De Xin Dang. *Formal analysis and investigation*: Seong Guk Chun. *Conceptualization, Methodology, Supervision, and Writing—review & editing*: In Ho Kim.

## Data Availability

The data that support the findings of this study are available from the corresponding author upon reasonable request.
